# The dual role of extracellular vesicles in vascular calcification: from molecular mechanisms to clinical translation

**DOI:** 10.3389/fcvm.2026.1815938

**Published:** 2026-05-07

**Authors:** Yuxia Zhang, Yujia Wang, Jian Lu, Xueqi Li, Zhiqing Chen, Xin Wang, Taotao Tang, Rining Tang

**Affiliations:** 1 School of Medicine, Southeast University, Nanjing, China; 2 Department of Nephrology, Nanjing Drum Tower Hospital, The Affiliated Hospital of Nanjing University Medical School, Nanjing, China

**Keywords:** vascular calcification, extracellular vesicles, pro-calcification, anti-calcification, therapeutic target

## Abstract

Vascular calcification (VC) is an active and regulated pathological process, which plays a central role in cardiovascular disease. Extracellular vesicles (EVs) are now recognized as crucial players in this pathology. EVs are nanoscale membrane vesicles secreted by cells. According to their biogenesis, they are mainly divided into exosomes, microvesicles and apoptotic bodies. They are rich in proteins, nucleic acids, lipids and other biologically active molecules. EVs play a dual role in VC. Regarding the pro-calcific role, EVs released by vascular smooth muscle cells (VSMCs), endothelial cells (ECs), and macrophages drive the phenotypic transformation of VSMCs by serving as nucleation cores for hydroxyapatite crystal deposition and by delivering pro-inflammatory and osteogenic signaling molecules. In addition to local effects, EVs also mediate long-distance intercellular communication. Together, these actions establish and amplify a pro-calcific microenvironment. In the aspect of anti-calcification, protective EVs can antagonize the osteogenic signaling pathway and maintain vascular homeostasis by delivering inhibitory microRNA (miRNA) (such as miR-126-5p, miR-133) and proteins (such as matrix Gla protein). The progress of VC depends on the balance between pro-calcific and anti-calcific EVs. Given their central position in pathology, EVs have become a highly promising source of new biomarkers, therapeutic intervention targets and drug delivery carriers. This review systematically summarizes the basic biological characteristics of EVs and the specific mechanisms underlying their dual regulatory roles in VC. It also discusses the challenges and future prospects for their clinical translation, thereby highlighting current knowledge gaps and outlining the exploratory nature of diagnostic and therapeutic strategies against VC.

## 1 Introduction

Vascular calcification (VC) is a serious vascular pathological change in common chronic diseases such as atherosclerosis, diabetes and chronic kidney disease (CKD). Epidemiological data show that the prevalence of VC rises sharply with age, as high as 52.6% to 95.36% in patients with end-stage renal disease, and is a primary contributor to their elevated cardiovascular mortality rates (1, 2). Its essence is the abnormal deposition of calcium and phosphate in the blood vessel wall in the form of hydroxyapatite. This process is not passive degradation, but an active and highly regulated biological event similar to bone development. According to the site of occurrence, it is mainly divided into intimal calcification (closely related to the instability of atherosclerotic plaques) and medial calcification (leading to increased arterial stiffness, which is an independent predictor of cardiovascular events) (3). The pathogenesis of VC is complex, involving the interplay of multiple factors, including the osteogenic phenotypic transformation of vascular smooth muscle cells (VSMCs), extracellular matrix remodeling, dysregulation of calcium and phosphate metabolism, and chronic inflammation (4). However, the detailed pathogenesis remains elusive, and effective clinical interventions are still lacking.

In recent years, Extracellular vesicles (EVs), as key carriers of intercellular communication, have been found to play a central role in the regulatory network of VC. On the one hand, under pathological stimulation, EVs released by a variety of cells, such as VSMCs, endothelial cells (ECs), macrophages, can serve as the initial nucleation site of microcalcification, and by delivering specific proteins (such as Annexins, alkaline phosphatase), nucleic acids [such as pro-calcific microRNA (miRNA)] and lipids, actively initiate and accelerate the deposition of hydroxyapatite crystals (5, 6). In addition, EVs also drive and amplify the calcification process by building complex local and long-distance intercellular communication networks (such as “bone-vascular axis”) to promote inflammation and osteogenesis signals (7). Notably, compared with the widely studied pro-calcific effect, the protective function of EVs in antagonizing calcification and its mechanism (such as the delivery of protective miRNA, calcification inhibitor protein, etc.) remains insufficiently studied and poorly understood. Understanding the dynamic balance between these pro- and anti-calcific effects is crucial for fully elucidating the pathological nature of VC.

This review aims to systematically outline the dual regulatory role of EVs in VC. We will introduce in detail the molecular mechanism and network of EVs as “pro-calcific messengers”. Secondly, we will summarize the current evidence supporting their anti-calcific function, although limited. The discussion also covers the potential and challenges of using EVs as new biomarkers, therapeutic targets, and drug delivery systems. Through systematic summary and outlook, this review aims to provide new ideas for the pathogenesis research of VC and the development of clinical intervention strategies.

## 2 Basic biology of extracellular vesicles

EVs are lipid-bilayer particles actively released by cells, and have emerged as key mediators of intercellular crosstalk. Understanding their biological origin, molecular composition and functional modes is the basis for clarifying their dual role in VC.

### 2.1 Biogenesis and classification

EVs are not a homogeneous group. According to their biogenesis, size and cargo, they are mainly divided into three categories: exosomes (originating from the endosomal system, with a diameter of 30–150 nm), microvesicles (formed by direct budding from the plasma membrane, diameter 100–1,000 nm) and apoptotic bodies (generated during programmed cell death, diameter >1 μm) (8). The formation of exosomes involves mechanisms such as the endosomal sorting complex required for transport (ESCRT) to identify, sort and package cargo into multivesicular bodies, which ultimately fuse with the plasma membrane for release (9–11). The formation of microvesicles is closely related to cell activation, which is triggered by an increase in intracellular calcium ion concentration, leading to loss of membrane phospholipid asymmetry [such as exposure of phosphatidylserine(PS)] and cytoskeletal reorganization, thereby promoting plasma membrane budding (12, 13). Apoptotic bodies are large vesicles formed by budding of the plasma membrane during programmed cell death. Their formation undergoes strictly regulated morphological steps such as membrane blebbing, formation of apoptotic membrane protrusions, and final fragmentation into apoptotic bodies. The formation of apoptotic bodies begins with the reorganization of the cytoskeleton, and caspase family proteases play a core regulatory role in this process (14, 15).

The difference in biogenesis pathways directly determines the membrane characteristics, size and initial cargo composition of EVs, which constitutes the structural basis of their functional heterogeneity.

### 2.2 Molecular cargo and selective sorting

The core function of EVs depends on the rich bioactive molecules they carry, including proteins, nucleic acids and lipids. This cargo is not randomly packaged but is precisely loaded through selective sorting mechanisms, thereby endowing EVs with specific biological information. Proteins: including transmembrane proteins (such as CD9, CD63, CD81) that reflect cellular origin, as well as cytoplasmic and membrane-associated proteins that are specifically sorted during biogenesis. For example, sortilin regulates the loading of tissue-nonspecific alkaline phosphatase (TNAP) into EVs to mediate VC (16, 17). Nucleic acids: EVs are rich in various RNAs, especially miRNAs and mRNAs. These nucleic acids can be delivered to recipient cells to regulate their gene expression (18). Selective sorting mechanisms ensure that specific miRNAs are preferentially packaged into EVs (19). Lipids: constitute the EV membrane skeleton, and their composition (such as cholesterol, sphingolipids, PS) affects membrane fluidity, rigidity and signaling capacity (20). Lipid raft microdomains also play an important role in cargo sorting (21). The precise regulation of cargo sorting means that cells can actively alter the molecular composition of EVs according to their own state and environmental signals, thereby potentially altering the functional impact of EVs on recipient cells in a context-dependent manner.

### 2.3 General modes of action

Based on their specific biogenesis pathways and the molecular cargo they carry, EVs interact with target cells and the microenvironment through a variety of distinct but overlapping mechanisms. These modes constitute the fundamental framework for their roles in diverse physiological and pathological processes.

One primary mechanism involves EVs acting as extracellular signaling platforms without the need for internalization. Ligands or receptors displayed on their surface (such as integrins, Notch ligands, or antigen-presenting complexes) can directly interact with corresponding receptors on the target cell membrane, triggering downstream intracellular signaling cascades and rapidly altering the target cell state (22).

In addition to surface signaling, EVs actively deliver their functional cargo—such as proteins, mRNAs, miRNAs, and lipids—into the recipient cell's cytoplasm to reprogram its proteomic and transcriptomic profiles (18). This intracellular delivery is achieved either through direct lipid-bilayer fusion with the plasma membrane or via highly regulated internalization pathways. Rather than a simple, generalized endocytosis, EV uptake occurs through multiple specific routes, including clathrin- or caveolin-dependent endocytosis, macropinocytosis, lipid raft-mediated internalization, and phagocytosis by immune cells (8, 23). Following internalization, EVs can subsequently fuse with the endosomal membrane to completely release their contents into the cytosol (24).

Beyond signal transduction and cargo delivery, specific types of EVs (such as matrix vesicles and apoptotic bodies) serve as extracellular structural and biomineralization components. They can directly participate in tissue construction by acting as initial nucleation cores for biomineralization via the aggregation of calcium and phosphate ions (25), or anchor to the ECM via surface integrins to orchestrate matrix remodeling (26).

Together, these diverse modes—surface ligand-receptor interactions, varied active internalization routes coupled with membrane fusion, and structural support—form a highly orchestrated operational framework through which EVs communicate with recipient cells and remodel the tissue microenvironment.

EVs are a highly heterogeneous, dynamic and multifunctional communication system. Their biogenesis pathways determine their basic properties, and selective sorting mechanisms endow them with specific molecular instructions, thereby enabling EVs to influence cells and the microenvironment. Although there are clear differences in biogenesis, size, and content among the three types of EVs mentioned above, current research mostly refers to them collectively as “EVs”, without fully distinguishing the unique contributions of their subtypes in VC. This heterogeneity is not merely a taxonomic detail; it directly shapes EV functional roles in the calcification microenvironment. For example, exosomes are rich in specific miRNAs and signaling proteins and primarily mediate intercellular reprogramming. Microvesicles are larger and carry abundant bioactive molecules on their surface, making them more prone to act as nucleation sites for hydroxyapatite. Apoptotic bodies contain fragmented organelles and high calcium levels, giving them the strongest calcification-inducing capacity, although they may also release immunosuppressive signals (as discussed in Section 3). Nevertheless, most VC studies have not systematically isolated or functionally compared EV subtypes, so conclusions about “pro-calcific” or “anti-calcific” EVs may mix effects from different subtypes. Thus, understanding EV heterogeneity is essential for accurately deciphering their dual role in VC and represents a core challenge for future translational research.

## 3 Pro-calcific roles of EVs in VC

Currently, the mechanistic landscape of EV-mediated VC remains fragmented. Most studies have focused on isolated pathways, and a unified model integrating these signals is not yet available due to the lack of cross-pathway comparative studies. Nevertheless, the following core mechanisms have been repeatedly identified.

### 3.1 EVs as nucleation cores for microcalcification

Calcification begins inside or on the surface of EVs. These specialized EVs act as nucleation foci and initiate the mineralization process by gathering calcium ions and phosphate ions. This process is highly ordered and regulated. Under calcification stimuli such as hyperphosphatemia, PS exposure on the membrane of EVs released by VSMCs increases and combines with calcium ions, providing a site for the initial deposition of calcium phosphate crystals (27). Furthermore, EVs are enriched with various calcium-binding proteins. Annexin family members (e.g., Annexin A5 and A6), particularly abundant in matrix vesicles (MVs, a subtype of EVs involved in mineralization), bind to both PS and Ca^2+^. This interaction forms PS- and Ca^2+^-enriched microdomains on the inner membrane leaflet, serving as a scaffold for calcium phosphate crystal growth (25). At the same time, the activity of alkaline phosphatase (ALP) carried in EVs increases, and the hydrolysis of organic phosphate produces Pi, resulting in local supersaturation of the concentration of calcium and phosphorus ions in the EVs membrane microenvironment (28, 29). Subsequently, the initial calcium phosphate complex (such as amorphous calcium phosphate) is formed on the inside or surface of the EVs membrane. These initial mineralization foci are used as nucleation seeds to further guide the epitaxial growth of hydroxyapatite crystals. *In vitro* studies have demonstrated the mineralization potential of EVs. Specifically, EVs isolated from the aortas of calcified mice and subsequently incubated in a high-phosphate solution can initiate mineralization, a process quantifiable by measuring absorbance at 340 nm. The formation and maturation of calcification minerals produced by EVs could also be observed in collagen hydrogel (30).

The interaction between EVs and extracellular matrix components is central to their nucleation function. The extracellular matrix (ECM) serves as a physical scaffold. It influences where EVs localize, how long they persist, and whether they remain bioactive. Type I collagen (COL1) is the main component of vascular ECM. It has been shown that EV surface integrins interact with specific hexapeptide sequences (GFOGER) in COL1, anchoring EVs to the ECM and promoting calcified foci formation (26). Annexin proteins can also mediate the interaction between EV and COL1 (31). This interaction is crucial for the spatial positioning of calcification. In addition, the physical and biochemical characteristics of ECM will also affect the behavior of EVs. For example, EVs released from calcified VSMCs and deposited in ECM have unique proteomic characteristics. Unlike EVs isolated from cell culture medium, these ECM-EVs show higher calcium content and stronger ability to induce macrophage inflammation (32). This process from nucleation to crystal growth gradually expands and fuses the microcalcification foci originally limited to EVs, and eventually forms macroscopic calcification lesions.

### 3.2 EVs in establishing a pro-calcific microenvironment

Beyond anchoring nucleation sites through interactions with the ECM, EVs play a central role in establishing a pro-calcific microenvironment by modulating both the extracellular matrix and inflammatory responses. Their release and pathological potential are tightly regulated at the source. Various pathological stimuli can specifically influence the secretion of EVs and their cargo loading, transforming them into potent pro-calcific packages. For instance, in the context of diabetes, high glucose activates macrophages via the S100A9-Receptor for advanced glycation end products (RAGE) axis, prompting them to release EVs enriched with pro-inflammatory and osteogenic factors. These EVs possess high calcification potential and directly exacerbate intraplaque microcalcification (28). Similarly, nicotine induced NADPH oxidase 5 (Nox5)-dependent oxidative stress increases the secretion of EVs while reducing the clearance of VSMCs on EVs, resulting in the formation of a calcified microenvironment (27). More targeted research demonstrates that inhibiting the lipid kinase PIKFYVE (phosphoinositide kinase, FYVE-type zinc finger containing), which is involved in EV biogenesis within VSMCs, can specifically reduce the loading of key pro-calcific cargo such as TNAP into EVs. This intervention effectively attenuates arterial calcification in both *in vitro* and *in vivo* models (33). These findings indicate that modulating EV release or cargo sorting is sufficient to alter the pathological trajectory of the microenvironment. Once released into the microenvironment, EVs act through multiple synergistic pathways to create conditions that may favor a self-reinforcing cycle. In addition to their role in initiating mineral deposition (30), EVs influence the microenvironment by modulating immune cell behavior. For example, EVs deficient in Gla-rich protein (GRP) not only exhibit a higher propensity for calcification but also can induce an inflammatory response in macrophages, creating a vicious “calcification-inflammation” cycle in ECM (32).

EVs, then, are far from isolated calcification carriers. They appear to be important modulators of the microenvironment. From upstream release regulation to downstream multifactorial effects, EVs construct a positive feedback network. This network amplifies and solidifies the initial pathological stimuli, ultimately driving the irreversible progression of VC.

### 3.3 EV-mediated communication networks in VC

#### 3.3.1 Local intercellular crosstalk

In addition to acting as passive nucleation sites, EVs play a key role in VC progression by actively carrying signals and forming a local communication network. In this network, VSMCs are not isolated—they act as central hubs and interact with nearby ECs and immune cells, mainly through EVs. This multi-directional crosstalk significantly promotes and maintains the calcification process.

##### 3.3.1.1 EVs as messengers of VSMC self-amplification

VSMCs take the lead by using EVs for autocrine and paracrine signals, creating a self-reinforcing loop. Studies show that EVs from the conditioned medium of calcified VSMCs can trigger osteogenic transdifferentiation in healthy VSMCs, with molecules like miR-324-3p playing a key role (5, 34). This spread of phenotypic change is supported by the specific cargo in VSMC-derived EVs, which reflects a pro-calcific state—marked by high levels of calcium-binding Annexins (A2, A5, A6) and matrix metalloproteinase-2 (MMP-2), and low levels of inhibitors like Matrix Gla Protein (MGP) (6). In addition, stress factors such as oxidative damage promote the release of Ca^2+^-loaded EVs from VSMCs, and blocking the uptake of these EVs can reduce calcification in target cells (27). EVs can also carry regulators of cellular stress, such as the mitochondrial-associated membrane protein glucose-regulated protein 75 (GRP75), which promotes calcification by impairing mitochondrial function (35). Overall, the EVs released by calcified VSMCs can both initiate mineralization and transmit various molecules to affect healthy VSMCs, further expanding the scope of calcification.

##### 3.3.1.2 Bidirectional EV signaling between ECs and VSMCs

ECs and VSMCs communicate bidirectionally via EVs, which has emerged as a critical axis in both vascular homeostasis and the pathogenesis of calcification. EC-derived EVs carry regulatory cargo—such as the miR-143/145 cluster or circ_0008362—that can be taken up by VSMCs, steering them toward osteogenic differentiation under diabetic conditions (36, 37). Pathological conditions like aging, uremia, and hyperglycemia transform the endothelium into a source of pro-calcific EVs. These EVs, enriched with factors like bone morphogenetic proteins (BMPs), Annexins, or versican, can induce oxidative stress, mitochondrial dysfunction, and senescence in VSMCs, thereby directly linking endothelial injury to medial calcification (38–41). The consequences extend beyond phenotypic modulation, as this EV-mediated crosstalk also feeds back onto the endothelium. EVs from activated VSMCs can also affect endothelial function, possibly forming a vicious cycle that worsens vascular dysfunction and disease progression (42, 43).

##### 3.3.1.3 EVs-mediated immune cells-VSMCs interactions: driving the inflammation-calcification cycle

The local network extends to include immune cells, particularly macrophages, with which VSMCs engage in a synergistic crosstalk that fuels a pro-inflammatory, pro-calcific microenvironment. Macrophage-derived EVs are potent inducers of VSMC phenotypic switching, carrying effectors like S100A9, miR-32, and galectin-3 that not only promote osteogenic reprogramming and suppress autophagy, but also influence where calcification occurs within the vessel wall by guiding EV trafficking (44–46). In the setting of diabetes or lipopolysaccharide (LPS) stimulation, the EV cargo from activated or foam macrophages becomes heavily skewed toward pro-inflammatory proteins (cis-aconitate decarboxylase, plasminogen activator inhibitor-1, serum amyloid A3) and signaling molecules that activate extracellular signal-regulated kinase (ERK) and protein kinase B (Akt) pathways in VSMCs, thereby linking local inflammation to enhanced VSMC migration, adhesion, and osteoblastic transformation (47, 48). Immune cells can influence a variety of phenotypes and functions of VSMCs related to calcification through EVs. Crucially, this relationship is directional. For example, EVs rich in calcium and deficient in GRP can induce the inflammatory response of macrophages, thus forming a “calcification-inflammatory” cycle and amplifying pathological signals (32). Therefore, EVs serve as carriers that orchestrate the delivery of both inflammatory and pro-calcific signals, making the inflammatory microenvironment a hotbed for the continuous progression of VC.

##### 3.3.1.4 Involvement of other vascular wall cells via EVs

In addition to the above core cell types, other cells in the vascular wall, such as pericytes and fibroblasts, may also participate in the construction of calcification networks through EVs. Pericytes can also be triggered to differentiate into chondrocytes and osteoblasts (49, 50). In addition, pericytes may mediate calcification by secreting matrix molecules such as laminin, type X collagen, and tenascin (51). Evidence suggests that calcification is mediated by bidirectional interactions between pericytes and ECs (52, 53). Emerging studies show that pericytes also play a role in mediating intercellular communication by secreting EVs (54). Based on calcification-associated genes identified through genome-wide association studies (GWAS), Qian et al. performed polygenic scoring on all cell types in vascular single-cell sequencing data from diabetic patients. They found that VSMCs and fibroblasts had the highest scores, leading to the speculation that these are the primary cell types involved in VC. In diabetic patients, VSMCs in calcified anterior tibial arteries can transdifferentiate into fibroblast-/chondrocyte-like cells (55). Moreover, fibroblasts may promote ectopic mineralization by releasing pro-calcific apoptotic bodies that drive M2 macrophage polarization (56). In addition, pericytes and fibroblasts may also release regulatory EVs under specific stress, affecting the homeostasis of VSMCs. Although their roles via EVs are less defined, their potential participation underscores VC as a multicellular process.

#### 3.3.2 Systemic inter-organ communication

The progress of VC is not only limited to the local lesions of the vascular wall, but also the concentrated manifestation of systemic metabolism and inflammatory disorders at the vascular level. Beyond local actions, EVs also mediate long-distance inter-organ communication, offering a potential mechanism for VC as a systemic disease.

Evidence suggests that EVs from CKD patients serum, but not EV-depleted serum, can induce VSMC calcification, indicating a direct role for EVs (57). The types of circulating EVs are diverse and complex. In addition to the local release of EVs of calcified blood vessels, there may be a variety of EVs secreted by other distant organs to play a role. There is a close interaction between the bone and the vascular system, forming a complex “bone-vascular axis”. Bone metabolism disorders, especially bone loss associated with uremia, aging and menopause, are often accompanied by the intensification of VC. This contradictory phenomenon is called the “calcification paradox" (58). Research shows that EVs are the key messengers to mediate this remote communication. In the process of bone resorption, EVs from the aging bone matrix are released into the circulation. By transmitting miR-483-5p and miR-2861, these EVs exert dual effects: they divert the fate of bone marrow mesenchymal stem cells (MSCs) from osteogenic to adipogenic differentiation (exacerbating bone marrow adiposity and osteoporosis) and remotely promote calcification of VSMCs (7). This signal transmission mediated by EVs provides a new perspective for understanding the mechanism of osteoporosis and VC.

In metabolic dysfunction-associated fatty liver disease (MAFLD), steatotic hepatocytes release increased EVs. These EVs carry lectin galactoside-binding soluble 3-binding protein (Lgals3bp), a protein that, triggers a chondrogenic-like shift of VSMCs and accelerates VC (59). These observations raise the possibility of a “liver-vascular axis”, which may also extend to adipose tissue. EVs may transmit metabolic stress signals and affect the systemic vascular homeostasis. These findings emphasize that EVs, as systemic messengers, transmit pro-calcific signals between organs, providing a new perspective for understanding the multi-organ influence of VC.

The intestine, a major endocrine and immune organ, might also talk directly to blood vessels through EVs—forming what could be called a “gut-vascular axis”. Gut microbiota and their metabolites, intestinal endocrine cells, etc. may release EVs carrying specific signals into the portal and systemic circulation, modulating systemic immunometabolic homeostasis and thereby influencing VC. Supporting this hypothesis, recent studies have shown that Lactobacillus rhamnosus GG exacerbates CKD-related VC by remodeling the gut microbiota, increasing EV release, and activating the phosphoinositide 3-kinase (PI3K)/Akt pathway in VSMCs (60). EVs isolated from Bacteroides fragilis have been confirmed to enter the circulation. These EVs aggravate VC in type 2 diabetic mice by activating the stimulator of interferon genes (STING)—myocyte enhancer factor (Mef2d) pathway, upregulating Trib1 and Serpine1 expression, and inducing M2 macrophage polarization (61). Although direct evidence is still accumulating, based on the key role of the intestines in systemic inflammation and the absorption of minerals (such as phosphate), EVs may mediate “gut-vascular crosstalk” and play an important role in VC.

In summary, emerging evidence points to the possibility that remote inter-organ communication mediated by EVs contributes to a complex network of systemic diseases. EVs released by bones, liver, intestines and other organs in the pathological state are like “special envoys” entering the circulation, potentially transmitting local metabolic abnormalities, inflammation or ecological imbalance information to the vascular system and directly regulating the fate of vascular cells. This breaks the traditional view that VC is regarded as isolated vascular lesions, establishes its central position in multi-organ crosstalk, and provides revolutionary treatment ideas for intervention in VC from a whole-body perspective. The key pro-calcific EV subtypes, their cargo molecules, and biological effects are summarized in Table 1.

**Table 1:** Pro-calcific roles of EVs in VC.

Pathophysiological role	Source	Subtype(as reported)	Cargo	Target	Biological effects	Ref.
EVs as Nucleation Cores and Establishing Pro-Calcific Microenvironment	VSMCs	EVs	Calcium phosphate, TNAP	ECM	Aggregates Ca/P ions to drive nascent mineral formation via TNAP activity.	(30, 33)
	Mineralizing VSMCs	EVs/MVs	Annexins (e.g., AnxA6), TNAP/ALP, PS, GRP75	ECM (TypeIcollagen)	Induces hydroxyapatite nucleation and collagen mineralization via AnxA6/PS complexes and GRP75/TNAP activity.	(31, 35)
	Macrophages	EVs/MVs	S100A9, Annexin V, ALP	ECM/Plaques	Facilitates hydroxyapatite nucleation via PS-Annexin V-S100A9 complexes and the S100A9-RAGE axis.	(28, 45)
	Biomimetic Models	MVs	Annexin A5	PS/Ca-enriched membranes	Stabilizes vesicle membranes to facilitate intraluminal mineral formation.	(25)
Local intercellular crosstalk						
*VSMC Self-Amplification*	Dedifferentiated VSMCs	EVs	Calcium	VSMCs	Increases cytosolic calcium and Nox5-mediated ROS production.	(27)
	Calcifying VSMCs	Exosomes	miR-324-3p, let-7e-5p	VSMCs	Promotes calcification by altering IGF1R/PIK3CA/MAP2K1 pathways.	(5)
	VSMCs	MVs	Annexins (II,V,VI), CD63	VSMCs	Activates MAPK signaling and increases intracellular NOX-1/SOD-2/Ca²⁺.	(34)
*EC-VSMC*	High glucose ECs	EVs	circ_0008362	VSMCs	Promotes osteogenic differentiation by sponging miR-1251-5p to upregulate Runx2.	(36)
	Senescent ECs	Microvesicles	AnxA2, AnxA6, BMP2, Ca²⁺	VSMCs	Promotes osteoblastic transformation via transfer of bone-associated proteins.	(39)
	ECs	EVs	miR-143 / miR-145	VSMCs	Regulates VSMC contractile phenotype based on EC morphology.	(37)
	ECs	EVs	HMGB1, HMGB2	VSMCs	Induces SASP, upregulates VCAM-1, and enhances leukocyte adhesion.	(38)
	IS-treated ECs	Microvesicles	N/A	VSMCs	Promotes VC via pro-inflammatory gene modulation.	(40)
	Hyperglycemic ECs	Exosomes	Versican	VSMCs	Induces senescence and calcification by modulating mitochondrial function.	(41)
*Immune Cells-VSMCs*	Mineralizing VSMCs	EVs	Calcium, GRP	Macrophages	Induces macrophage inflammation via ECM-deposited EV signaling.	(32)
	VSMCs	EVs	N/A	Macrophages / Intima	Promotes intimal calcification via Galectin-3-mediated translocation in diabetes.	(44)
	Macrophages	EVs	miR-32	VSMCs	Promotes osteogenic differentiation by inhibiting autophagy (Mef2d/cGMP-PKG).	(46)
	LPS-macrophages	EVs	Cytokines, CAD, PAI-1, Saa3	VSMCs	Propagates inflammation/oxidative stress and induces osteogenic switch in atherosclerosis.	(47)
	Macrophage foam cells	EVs	Integrins (β1, α5)	VSMCs	Promotes VSMC migration/adhesion via integrin transfer and ERK/Akt activation.	(48)
*Other Vascular Cells*	PROCR^+^ fibroblasts	Apoptotic bodies	Calcium	ECM & Macrophages	Increases ECM stiffness and initiates M2 polarization for calcification.	(56)
Systemic Inter-Organ Crosstalk	Circulating (Serum)	sEVs	miRNAs (miR-16-5p, etc.)	VSMCs	Depletion of miRNAs accelerates VSMC phenotypic switching via VEGFA signaling in CKD.	(57)
	Aged bone matrix/Osteocytes	EVs	miR-2861 and Calcium	VSMCs	Delivers miR-2861 to VSMCs to stimulate RUNX2 expression and osteogenic transdifferentiation.	(7)
	Steatotic hepatocytes	EVs (exosome-enriched)	Lgals3bp	VSMCs & Macrophages	Induces VSMC osteochondrogenic switch and M1 macrophage polarization in MAFLD.	(59)
	Lactobacillus	Bacterial EVs	N/A	VSMCs	Upregulates osteogenic factors (RUNX2, BMP2) via PI3K/AKT pathway in CKD.	(60)
	Bacteroides fragilis	EVs	dsDNA	Macrophages	Exacerbates VC via Sting-mediated M2 polarization and Serpine1 upregulation.	(61)

Akt, protein kinase B; ALP, alkaline phosphatase; AnxA, annexin A; BMP2, bone morphogenetic protein 2; Ca/P, calcium/phosphate; CAD, cis-aconitate decarboxylase; cGMP-PKG, cyclic guanosine monophosphate–protein kinase G; CKD, chronic kidney disease; dsDNA, double-stranded DNA; ECM, extracellular matrix; ECs, endothelial cells; ERK, extracellular signal-regulated kinase; EVs, extracellular vesicles; GRP, Gla rich protein; GRP75, glucose-regulated protein 75; HMGB, high mobility group box; IGF1R, insulin-like growth factor 1 receptor; IS, indoxyl sulfate; Lgals3bp, lectin galactoside-binding soluble 3-binding protein; LPS, lipopolysaccharide; MAFLD, metabolic dysfunction-associated fatty liver disease; MAP2K1, mitogen-activated protein kinase kinase 1; MAPK, mitogen-activated protein kinase; Mef2d, myocyte enhancer factor 2D; miR/miRNAs, microRNA/microRNAs; MVs, matrix vesicles/microvesicles; NOX, NADPH oxidase; PAI-1, plasminogen activator inhibitor-1; PI3K, phosphoinositide 3-kinase; PIK3CA, phosphatidylinositol-4,5-bisphosphate 3-kinase catalytic subunit alpha; PROCR, protein C receptor; PS, phosphatidylserine; RAGE, receptor for advanced glycation end products; ROS, reactive oxygen species; RUNX2, Runt-related transcription factor 2; S100A9, S100 calcium-binding protein A9; Saa3, serum amyloid A3; SASP, senescence-associated secretory phenotype; sEVs, small extracellular vesicles; SOD-2, superoxide dismutase 2; STING, stimulator of interferon genes; TNAP, tissue-nonspecific alkaline phosphatase; VC, vascular calcification; VCAM-1, vascular cell adhesion molecule 1; VEGFA, vascular endothelial growth factor A; VSMCs, vascular smooth muscle cells.

## 4 Anti-calcific roles of EVs in VC

### 4.1 Core molecular mechanisms of anti-calcific EVs

Current research has identified several distinct mechanisms by which anti-calcific EVs may counteract VC. These can be conceptually grouped into a collaborative defense network, ranging from direct inhibition of crystal formation to modulation of the cellular microenvironment.

The first line of defense is direct “deactivation” and neutralization. This represents the most immediate physicochemical defense, aimed at removing or neutralizing calcification “seeds”. Protective EVs can deliver key calcification-inhibitory proteins to the calcification microenvironment, such as functional MGP, GRP, and Fetuin-A. These proteins efficiently bind calcium and phosphate ions, or directly bind to nascent hydroxyapatite crystals, thereby preventing further crystal growth and aggregation (32, 62, 63). For example, in patients with CKD, the deficiency of functional GRP and Fetuin-A in circulating EVs is directly associated with an enhanced pro-calcific capacity (32). The second line of defense is cellular “reprogramming” and phenotype maintenance. This defense aims to preserve the normal contractile phenotype of VSMCs and prevent their transdifferentiation into osteoblast-like cells. For example, in the CKD model, the miRNA transcriptome analysis of circulating small EVs found that the depletion of miRNAs such as miR-16-5p, miR-17-5p, miR-20a-5p and miR-106b-5p promoted VC. These miRNAs inhibit the osteogenic phenotypic switching of VSMCs by targeting the vascular endothelial growth factor A(VEGFA) signaling pathway (57). Protective EVs achieve this by delivering specific protective miRNAs. For instance, miR-126-5p delivered by ECs-derived EVs inhibits the BMP/ Sma- and Mad-related protein (Smad)1/5/9 signaling pathway by targeting bone morphogenetic protein receptor type 1B (BMPR1B), effectively blocking high glucose-induced osteogenic differentiation of VSMCs (64). Similarly, engineering EVs to be loaded with miR-133, which targets the key osteogenic transcription factor Runt-related transcription factor 2 (Runx2), successfully inhibited chondrogenic transdifferentiation of VSMCs and reduced calcification in an atherosclerosis model (65). The miR-381-3p delivered by stem cell-derived EVs inhibits the osteogenic shift in VSMCs by targeting nuclear factor of activated T-cells 5 (66). The next line involves microenvironment “repair” and anti-inflammation. VC is closely linked to a chronic inflammatory microenvironment; therefore, suppressing inflammation is a crucial aspect of anti-calcification. Protective EVs can break the “inflammation-calcification” vicious cycle by transferring anti-inflammatory molecules. For example, miR-17-5p carried by macrophage-derived EVs may mitigate the inflammatory response and attenuate VSMC osteogenesis by inhibiting TGF-β (transforming growth factor beta) signaling (67). Furthermore, MSC-EVs can deliver the long non-coding RNA NONHSAT084969.2, which alleviates high phosphate-induced VSMC calcification by modulating the nuclear factor kappa B (NF-κB) signaling axis (68).

Systemic regulation represents another layer of defense. It primarily engages endogenous repair and regulatory systems—notably EVs from stem cells, particularly mesenchymal stem cells (MSCs). These EVs do more than deliver protective molecules. Through their inherent immunomodulatory, antioxidant, and pro-vascular repair paracrine effects, they reshape both the systemic and local microenvironment, creating an internal milieu that resists ectopic calcification (66, 69). In CKD-VC models, MSC-EVs have demonstrated comprehensive protective effects against calcification and apoptosis (66, 68). The main anti-calcific mechanisms, including direct inhibition, cellular reprogramming, and systemic regulation, are summarized in Table 2.

**Table 2:** Anti-calcific roles of EVs in VC.

Pathophysiological role	Source	Subtype(as reported)	Cargo	Target	Biological effects	Ref.
Anti-calcific mechanisms						
*Autocrine Defense & Biogenesis Control*	VSMCs (GFOGER peptide-treated))	EVs	Normalized proteins: Ppm1f (phosphatase), Csnk2a2/CKII (casein kinase II), Tbk1 (kinase)	ECM	Inhibits pro-calcifc EV release via modified intracellular protein sorting.	(26)
	VSMCs	EVs	GRP	VSMCs	Prevents osteogenic differentiation and limits intraluminal calcium accumulation.	(32)
	VSMCs (EGFR inhibitor-treated)	EVs	N/A	EV biogenesis pathway	Prevents biogenesis and release of pro-calcifc EVs via EGFR inhibition.	(70)
*Local Paracrine Compensation*	Macrophages	EVs	miR-17-5p	VSMCs	Attenuates VSMC osteogenic differentiation as a therapeutic target.	(67)
	HUVECs (AGEs-treated)	sEVs	miR-126-5p	VSMCs	Targets BMPR1B and inhibits smad1/5/9 signaling to suppressVSMC osteogenic differentiation and calcification.	(64)
*Systemic Protective EVs*	Circulating (Healthy serum)	EVs	Fetuin-A, MGP, GRP	VSMCs	Prevents crystal growth via physiological loading of calcification inhibitors.	(62)
	Circulating (Healthy serum)	sEVs	miR-16-5p, miR-17-5p, miR-20a-5p, miR-106b-5p	VSMCs	Prevents VSMC phenotypic switching by suppressing VEGFA-VEGFR2 signaling in CKD.	(57)
*Stem Cell-Derived Therapeutics*	BMSCs	Exosomes	miR-381-3p	VSMCs	Attenuates high phosphorus-induced osteogenic differentiation in CKD.	(66)
	BMSCs	Exosomes	Downregulated lncRNA NONHSAT 084969.2	VSMCs	Alleviates high phosphorus-induced VSMC calcification via paracrine effects.	(68)

AGEs, advanced glycation end products; BMPR1B, bone morphogenetic protein receptor type 1B; BMSCs, bone marrow mesenchymal stem cells; ECM, extracellular matrix; EGFR, epidermal growth factor receptor; EVs, extracellular vesicles; GFOGER, Gly-Phe-Hyp-Gly-Glu-Arg; GRP, Gla rich protein; HUVECs, human umbilical vein endothelial cells; lncRNA, long non-coding RNA; MGP, matrix Gla protein; miR, microRNA; sEVs, small extracellular vesicles; Smad, Sma- and Mad-related protein; VEGFA, vascular endothelial growth factor A; VEGFR2, vascular endothelial growth factor receptor 2; VSMCs, vascular smooth muscle cells.

### 4.2 The dynamic balance and regulation between pro- and anti-calcific EVs

The progression of VC essentially reflects the breaking of the dynamic balance between pro-calcific and anti-calcific EVs. This balance is regulated by multiple factors, including cellular origin, environmental stimuli, and molecular cargo. Under pathological states such as CKD or diabetes, pro-calcific EVs tend to dominate. For instance, EVs shed by senescent endothelial cells or steatotic hepatocytes carry pro-inflammatory and osteogenic factors like Lgals3bp, directly promoting VSMCs calcification (59). In contrast, anti-calcific EVs, typically from healthy endothelial cells or stem cells, counteract these effects by delivering protective miRNAs (e.g., miR-126-5p) and proteins such as matrix Gla protein (MGP) (64). This imbalance may lead to the aggravation of VC. In CKD, the miRNA spectrum of circulating EVs shows the depletion of protective miRNAs, while promoting calcification signals upregulation (57). Restoring this balance represents a potential therapeutic strategy. External interventions such as GFOGER peptide or drug BGP-15 can restore balance by modifying EVs goods, such as reducing osteogenic markers and increasing anti-calcific molecules such as MGP (26, 63). Membrane proteins such as Caveolin-1 play a key role in the formation of EVs and the regulation of mineralization potential. Caveolin-1 is not only an important part of cell membrane, but also participates in the biosynthesis and release of EVs. Studies show that the inhibition of Caveolin-1 can significantly reduce the generation of pro-calcific EVs, thus reducing the risk of VC (70). Understanding this dynamic balance is conducive to the development of targeted therapies, such as using engineered EVs (such as EVs loaded with miR-133) to enhance anti-calcific effect, while inhibiting the release of pro-calcific EVs (65). Overall, maintaining a balance between pro-calcific and anti-calcific EVs is a key strategy for preventing and treating VC.

EVs play a dual role as both the “spear” and the “shield” in VC. Current research on anti-calcific EVs remains relatively limited. A deeper understanding of the mechanisms of action of anti-calcific EVs and their balanced relationship with pro-calcific EVs will provide a conceptual foundation for diagnostic and therapeutic strategies aimed at restoring vascular homeostasis.

## 5 Clinical translation of EVs in VC

**Figure 1:**
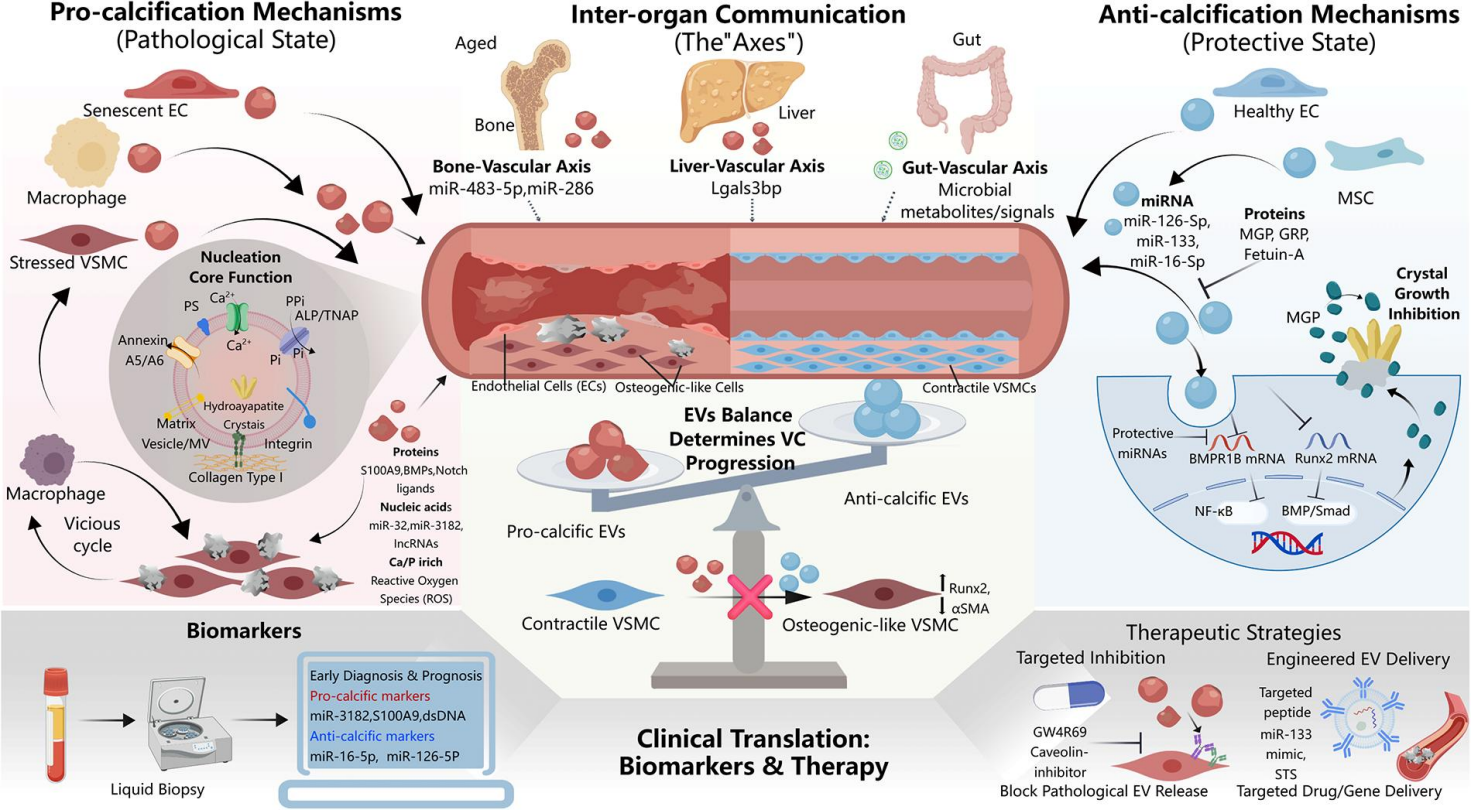
Schematic illustrating the dual role of extracellular vesicles (EVs) in vascular calcification. Left side: In pathological environment, stressed cells release EVs that promote mineral nucleation, transfer pro-calcific RNAs/proteins, induce VSMC osteogenic differentiation, and enable systemic crosstalk with bone, liver, and gut. Right side: In physiological state, EVs maintain homeostasis by delivering calcification inhibitors (MGP, Fetuin-A) and protective miRNAs. Imbalance between pro-calcific and anti-calcific EVs drives calcification. Bottom: Clinical applications include EV-based biomarkers for risk stratification and engineered EVs for targeted therapy. Created with BioGDP.com (80). ALP, alkaline phosphatase; BMP, bone morphogenetic protein; EC, endothelial cell; MGP, matrix Gla protein; MSC, mesenchymal stem cell; PS, phosphatidylserine; VSMC, vascular smooth muscle cell; EV, extracellular vesicles.

Based on the detailed molecular mechanisms outlined above, EVs have attracted interest for their possible translational potential in the clinical diagnosis and treatment of VC. Their proposed core value includes the possibility of serving as a non-invasive biomarker that reflects early changes of the disease, functioning as intervenable targets, and potentially being a direct therapeutic tool. However, it should be emphasized that most of these applications remain at an early, exploratory stage. A schematic overview of the dual role of EVs in VC pathogenesis and its therapeutic implications is presented in Figure 1.

### 5.1 EVs as diagnostic and prognostic biomarkers

EVs have garnered widespread attention in the study of VC biomarkers, and their potential for clinical translation is gradually emerging. Changes in the molecular cargo of EVs in the blood or tissue can reflect the early pathogenesis of VC, providing a new molecular basis for early diagnosis and prognosis of the disease (71). For instance, levels of miR-16-5p, miR-17-5p, and miR-106b-5p in plasma EVs are significantly decreased in CKD patients. These miRNAs, known to regulate VSMC osteogenic differentiation by targeting the VEGFA pathway, exhibit area under the curve (AUC) values of 0.74–0.77 for predicting VC, suggesting a possible diagnostic value that warrants further validation in larger cohorts (57). In addition, the expression of miR-3182 in EC-EVs induced by hyperphosphatemia is upregulated, and the calcification of VSMCs is promoted through the calcium signaling pathway, raising the hypothesis that the miRNA profile of EVs could potentially serve as an early warning indicator of VC (72). In patients with diabetic VC, the level of miR-126-5p carried by plasma EVs is negatively correlated with the aortic calcification score (64). Clinical sample analysis also found that EVs of diabetic VC patients are rich in double-stranded DNA (dsDNA), which activates macrophage M2 polarization through the STING-Mef2d pathway and further promotes the calcification process (61). These findings provide a theoretical basis for the development of liquid biopsy technology based on EVs. Further verification of elevated pro-calcific cargo and reduced anti-calcific substances in EVs may lead to non-invasive diagnostic tools for early VC.

### 5.2 EVs as therapeutic targets

Therapeutic strategies targeting EVs have shown some encouraging results in preclinical models of VC. Firstly, inhibiting the generation and release of EVs ameliorates VC. Inhibiting sphingomyelinase (such as GW4869) can reduce the release of EVs in the CKD model, leading to attenuated aortic calcification and reversal of osteogenic marker expression (57). Additionally, targeting key EV-associated proteins (e.g., via siRNA-mediated knockdown of caveolin-1 or epidermal growth factor receptor) can block the biogenesis of pro-calcific EVs and significantly ameliorate VC in animal models (70). Secondly, blocking the pathological functions of EVs represents another approach: intervening at key nodes through which pro-calcific EVs exert their effects. The hexapeptide sequence GFOGER in COL1 mediates its binding to integrins on EVs. Synthetic GFOGER peptide can act as a competitive inhibitor, disrupting EV-collagen interaction and thereby inhibiting calcification *in vitro* (26). It is worth noting that the S100A9-RAGE axis promotes the release of pro-calcific EVs from macrophages. Targeting this axis with inhibitors (e.g., RAGE antagonists) reduces microcalcification plaque formation in mouse models (28). These studies reveal multiple mechanisms of EVs as therapeutic targets, including the modulation of inflammation, oxidative stress and cell phenotypic transformation. While these preclinical findings are promising, their translation to human therapy remains a long-term goal.

### 5.3 EVs as potential drug delivery vehicles: dual therapeutic strategies

The inherent targetability, high biocompatibility, and low immunogenicity of EVs make them ideal vehicles for targeted drug delivery (73). To align with the dual role of EVs in the pathogenesis of VC, therapeutic strategies utilizing EVs as delivery platforms can be logically categorized into two main conceptual levels: inhibiting pro-calcific pathways and promoting protective or regenerative signaling.

#### 5.3.1 EV-based strategies to inhibit calcification

The first strategy involves engineering EVs to deliver specific inhibitors, such as non-coding RNAs or chemical drugs, directly to the calcification sites to block osteogenic pathways. For instance, researchers have utilized engineered EVs loaded with miR-133 to target and suppress key osteogenic transcription factors like Runx2 in VSMCs, significantly reducing calcification in atherosclerosis models (65). Beyond genetic cargo, EVs can also deliver chemical therapeutics. A notable study utilized grapefruit-derived EVs to deliver sodium thiosulfate (STS). By modifying the EV surface with a hydroxyapatite-binding peptide (HABP), these EVs specifically accumulated in calcified vessels, effectively inhibiting VC while successfully avoiding the systemic toxicity typically associated with free STS administration (74). However, such studies are proof-of-concept and have not yet been tested in humans.

#### 5.3.2 EV-based strategies to promote protective or regenerative signaling

The second strategy shifts the focus from direct inhibition to restoring vascular homeostasis and promoting tissue repair. Stem cell-derived EVs, particularly those from mesenchymal stem cells (MSC-EVs), are highly valued for this purpose. MSC-EVs inherit the anti-inflammatory, immunomodulatory, pro-regenerative, and pro-angiogenic properties of their parent cells (69). Recent studies have demonstrated that MSC-EVs can ameliorate VC in animal models by delivering bioactive protective signals that modulate macrophage polarization and reduce vascular oxidative stress (66, 68). As cell-free alternatives to traditional stem cell therapy, MSC-EVs and their engineered derivatives can reconstruct a protective microenvironment within the vascular wall, making them a promising area of investigation for VC therapy. Nevertheless, the efficacy and safety of such approaches in humans remain to be established. Additionally, plasma EVs from diabetic patients enriched in miR-126-5p are negatively correlated with aortic calcification scores (64), raising the hypothesis that restoring such protective miRNA profiles via engineered EVs could be explored, but this remains highly speculative at present.

### 5.4 Critical challenges and forward-thinking strategies for EV-based VC therapy

While EVs have generated considerable interest as VC therapeutics, it is important to recognize that their clinical translation remains distant and faces a set of interconnected, VC-specific hurdles that extend beyond general drug delivery challenges. The following discussion outlines these obstacles, many of which have only begun to be addressed in proof-of-concept studies.

#### 5.4.1 The targeting-biodistribution paradox in the calcified niche

A primary VC-specific challenge is achieving sufficient delivery to the often deep-seated and heterogeneous calcific lesions. Following systemic administration, non-targeted EVs undergo rapid clearance (75), leading to poor accumulation at the vascular site. While surface engineering with ligands like HABP offers a calcification-centric solution (74), the evolving and densely packed nature of advanced calcific plaques can limit ligand accessibility. Future strategies must therefore evolve towards multi-stage targeting or “smart” EVs designed to respond to the unique VC microenvironment (e.g., local high phosphate or protease activity) for precise, spatiotemporal release (76).

#### 5.4.2 Cargo-loading for VC-specific therapeutics

Loading strategies must be optimized for VC-relevant cargo. Delivering small molecule inhibitors like sodium thiosulfate requires methods that prevent premature leakage while maintaining EV integrity. For nucleic acids like miR-133 that target osteogenic drivers (e.g., Runx2) (65), loading techniques must ensure sufficient payload protection and efficient cytosolic delivery in the often senescent or stressed VSMCs of the calcified niche. The choice between endogenous and exogenous loading thus hinges on preserving the functional EV properties essential for navigating this pathological microenvironment.

#### 5.4.3 The VC-specific triad: safety, scalability, and regulation

Safety assessments for VC must consider chronic administration in patients with underlying conditions like CKD, requiring rigorous evaluation of potential off-target effects on bone metabolism and mineral homeostasis. Scalable manufacturing must address batch consistency while ensuring that the final EV product retains its anti-calcific potency, a metric that needs standardization per MISEV guidelines (77). From a regulatory standpoint, VC presents a unique challenge as a chronic, structural cardiovascular complication. Defining clinical endpoints and developing a regulatory pathway for an EV-based therapy aimed at halting or reversing VC, rather than treating an acute illness, requires novel frameworks and close collaboration with agencies, as outlined in recent consensus papers (78, 79).

#### 5.4.4 Future perspectives: towards VC-precision medicine

Looking forward, one possible direction is the development of integrated, VC-precision platforms. This could include engineering modular EVs capable of sequential targeting and stimulus-responsive drug release, as well as establishing VC-specific pharmacokinetic/pharmacodynamic models to rationalize dosing. Furthermore, leveraging EV-based biomarkers (discussed in Section 5.1) as companion diagnostics could enable patient stratification, ensuring that these advanced therapies are directed to those most likely to benefit. Ultimately, conquering VC through EV therapy demands a concerted, cross-disciplinary effort to co-develop the biological solutions and the tailored translational frameworks they require.

## 6 Discussion

EVs appear to be important regulators of VC, contributing to a balance between pro-calcific and anti-calcific signals. When pathological conditions intervene, that balance fractures. EVs acquire both mineral-nucleating and signal-transducing functions, helping build a pro-inflammatory, osteogenic niche and enabling cross-talk across local and systemic networks. Conversely, protective EVs from specific cellular sources deliver cargo that counteracts these processes, preserving vascular homeostasis. This duality underscores EVs not merely as disease byproducts but as active determinants of VC fate. That is why EVs are now viewed as translational assets with two distinct uses: their molecular signatures can be mined for early, non-invasive diagnostic markers, and their innate properties make them appealing as therapeutic targets and drug delivery vehicles (Figure 1).

Despite this central role, a number of conflicting findings and limitations should be acknowledged. The functional classification of EVs as either “pro-calcific” or “anti-calcific” depends heavily on cellular context and disease stage, making a rigid binary distinction potentially misleading. For example, while most studies report that macrophage-derived EVs promote VSMC osteogenesis, the precise miRNA and protein signatures vary with the polarization state of the parent macrophages (81), and some EV subpopulations may even carry protective signals (67)—in contrast to the pro-calcific effects reported under other conditions (46, 47). A deeper concern is that the majority of mechanistic studies rely on *in vitro* culture systems where EV isolation and dosing do not accurately mirror physiological conditions. Supraphysiological concentrations of exogenous EVs commonly used in functional assays may overwhelm endogenous buffering systems, leading to exaggerated or artifactual outcomes. The marked heterogeneity of EV populations also remains a major confounder. Many studies pool all small EVs without subtype separation, yet exosomes, microvesicles and apoptotic bodies differ in cargo composition and functional capacity. Beyond experimental heterogeneity, the very nomenclature of EV subtypes has been a source of confusion. Many studies still use the term “exosome” to refer to any small EV preparation without validating endosomal origin, while others use “microvesicle” or “apoptotic body” based solely on size or isolation method. This lack of terminological rigor hampers cross-study comparison and reproducibility. Following the MISEV2023 guidelines, we encourage the field to adopt neutral, operational terms (e.g., “small EVs” vs. “medium/large EVs”) unless specific biogenetic markers are demonstrated. For calcification research in particular, reporting standardized parameters such as particle-to-protein ratio, marker enrichment and functional activity per particle will greatly enhance data comparability across laboratories. The lack of standardized protocols for EV isolation, quantification, and characterization specifically tailored to calcification research further complicates cross-study comparisons and data reproducibility. These caveats do not invalidate the core findings but emphasize the need for well-controlled studies that fully account for EV heterogeneity and physiological relevance.

A key unanswered question is the quantitative contribution of anti-calcific EVs *in vivo*. While exogenous administration of protective EVs (for instance, from MSCs or ECs) attenuates experimental VC (64, 66, 68), the relative abundance and functional impact of endogenous anti-calcific EVs under physiological or pathological conditions remain largely unknown. Do circulating protective EVs exist at sufficient concentrations to meaningfully counteract their pro-calcific counterparts? Preliminary evidence from CKD patients suggests that the balance shifts markedly toward pro-calcific EVs, as reflected by depletion of protective miRNAs such as miR-16-5p and miR-17-5p in circulating EV fractions (57). However, direct quantitative comparisons—for example, the ratio of pro-calcific to anti-calcific EV particle numbers or the relative concentrations of their functional cargo—have not been systematically performed. The absence of subtype-specific EV markers makes such measurements technically demanding, but emerging single-vesicle analysis technologies may offer a feasible path forward. These considerations naturally raise the question: how is the balance between pro- and anti-calcific EVs actually regulated? It must be acknowledged that the anti-calcific EV section in the current literature—and consequently in this review—remains less developed compared to the pro-calcific counterpart. This disparity reflects the relative infancy of research on protective EV mechanisms, which have been hampered by the lack of robust *in vivo* models for tracking endogenous anti-calcific EV activity and the absence of consensus on what constitutes a “protective” EV signature. We therefore advocate for dedicated efforts to identify and validate anti-calcific EV cargoes, to elucidate their cellular sources under homeostasis, and to develop scalable methods for producing potent protective EV formulations. Until such studies emerge, the therapeutic promise of anti-calcific EVs will remain incompletely realized.

We propose that the equilibrium between pro-calcific and anti-calcific EVs is governed by at least three interconnected layers of regulation. The cellular source and metabolic state determine baseline EV cargo. Under pathological stressors such as high phosphate, oxidative stress, or inflammatory cytokines, cells rewire their EV sorting machinery to preferentially load pro-calcific cargo while reducing protective cargo such as MGP or Fetuin-A. Microenvironmental cues—local calcium and phosphate concentrations, pH, and inflammatory mediators—directly modulate EV release rates and composition via signaling cascades including NF-*κ*B, BMP/Smad, and PI3K/Akt. Finally, recipient cell competence dictates whether a given EV signal turns out to be pro-calcific or anti-calcific. VSMCs with high expression of specific receptors or robust endocytic machinery may be particularly susceptible to pro-calcific EV uptake, whereas those with efficient lysosomal degradation pathways might neutralize harmful EV cargo. Thus, restoring balance in VC is not simply a matter of increasing the absolute number of anti-calcific EVs, but rather requires re-establishing the normal regulatory logic of EV biogenesis, cargo sorting, and target cell responsiveness.

EVs have been implicated as potential messengers mediating the calcification paradox (7). Notably, osteoclast-like cells derived from the monocyte/macrophage lineage have been detected within calcified arterial walls (82). The imbalance between osteoblast-like and osteoclast-like cell regulation may also play an important role in the occurrence and development of VC regulated by EVs. However, the other half of the bone-vascular axis, namely osteoclast-mediated bone resorption and its core regulatory pathways, remains largely unexplored in EV-mediated VC. Osteoclasts are the only cells responsible for physiological bone resorption, and their differentiation is tightly controlled by the receptor activator of nuclear factor kappa-B ligand (RANKL)/osteoprotegerin (OPG) signaling axis, which also directly promotes VSMC osteogenic differentiation and VC (83). Given that osteoclasts originate from the monocyte/macrophage lineage and that macrophage-derived EVs are known regulators of VC, a natural question arises: when macrophages differentiate into osteoclast-like cells under RANKL stimulation, do their EVs acquire new functions that influence VC? Emerging evidence shows that osteoclast-derived EVs carry RANK and a variety of proteins and growth factors, which couple bone resorption to formation within the bone microenvironment (84, 85). Furthermore, EV composition changes dynamically during RANKL-induced osteoclast differentiation (86). Although no direct evidence links osteoclast-derived EVs to VC, the established pathophysiological connection between bone resorption and VC—exemplified by the calcification paradox and the RANKL/OPG axis—supports further investigation into this underexplored direction.

Several distinct challenges remain to be emphasized on the path to clinical translation of EV-based strategies. The chronic progressive nature of VC demands long-term safety evaluation, with particular attention to potential off-target effects on physiological bone metabolism, since EVs engineered to carry anti-calcific molecules might inadvertently interfere with normal skeletal mineralization (85). The heterogeneity of VC itself—intimal vs. medial calcification, microcalcification vs. macrocalcification—likely requires tailored EV formulations. For instance, microcalcification within vulnerable plaques may benefit from local delivery of anti-inflammatory EVs, whereas medial calcification in CKD may necessitate systemic correction of mineral ion balance via EV-mediated cargo delivery. Manufacturing and regulatory hurdles are equally substantial: large-scale production of clinical-grade EVs demands robust quality control metrics including particle count, cargo integrity, and potency assays, while the regulatory pathway for EV therapeutics remains under active development. Despite these challenges, we believe that EV-based approaches hold transformative potential, particularly when combined with patient stratification using EV-based biomarkers as companion diagnostics.

In summary, the dual role of EVs in VC—serving both as propagators of calcification and as carriers of protective signals—underscores their centrality in this pathological process. The balance between pro-calcific and anti-calcific EVs is dynamically regulated by cellular source, microenvironmental cues, and recipient cell competence, offering multiple nodes for therapeutic intervention. Moving from bench to bedside, addressing conflicting evidence, quantifying the *in vivo* contribution of anti-calcific EVs, and overcoming manufacturing and regulatory hurdles are essential steps. With advances in single-vesicle analysis, standardized isolation protocols, and rationally engineered EV formulations, the field is poised to translate the dual role of EVs into precise diagnostic biomarkers and effective therapies for VC. Nevertheless, current knowledge remains fragmented and lacks a unified model that integrates the key pro-calcific and anti-calcific pathways. Thus, more systematic and integrative studies are urgently needed to move the field forward.
